# Experiences of teachers and community-based health workers in addressing adolescents’ sexual reproductive health and rights problems in rural health systems: a case of the RISE project in Zambia

**DOI:** 10.1186/s12889-023-15199-5

**Published:** 2023-02-15

**Authors:** Kunda chilambe¹, Chama Mulubwa, Joseph Mumba Zulu, Malizgani Paul Chavula

**Affiliations:** 1 Department of Health Policy and Education, School of Public Health, University of Zambia, Lusaka, P.O Box 50110, Zambia; 2 Department of Epidemiology and Global Health, Umea University, Umea, 90187 Sweden

**Keywords:** Sexual, Reproductive Health, Rights, Comprehensive Sexuality Education, RISE, Rural health systems

## Abstract

**Background:**

Adolescents in low-and-middle-income countries like Zambia face a high burden of sexual, reproductive, health and rights problems including coerced sex, teenage pregnancies, and early marriages. The Zambia government through Ministry of Education has integrated comprehensive sexuality education (CSE) in the education and school system to contribute towards addressing Adolescents sexual, reproductive, health and rights (ASRHR) problems. This paper sought to explore teachers and community based health workers (CBHWs)’ experiences in addressing ASRHR problems in in rural health systems in Zambia.

**Methodology:**

The study was conducted under Research Initiative to Support the Empowerment of Girls (RISE) community randomized trial that aims to measure the effectiveness of economic and community interventions in reducing early marriages, teenage pregnancies, and school dropout in Zambia. We conducted qualitative 21 in-depth interviews with teachers and CBHWs involved in the implementation of CSE in communities. Thematic analysis was used to analyse teachers and CBHWs´ roles, challenges, and opportunities in promoting ASRHR services.

**Results:**

The study identified teachers and CBHWs roles, and challenges experienced in promoting ASRHR and suggested strategies to enhance delivery of the intervention. The role of teachers and CBHWs in addressing ASRHR problems included mobilizing and sensitizing the community for meetings, providing SRHR counseling services to both adolescents and guardians, and strengthening referral to SRHR services if needed. The challenges experienced included stigmatization associated with difficult experiences such as sexual abuse and pregnancy, shyness among girls to participate when discussing SRHR in the presence of the boys and myths about contraception. The suggested strategies for addressing the challenges included creating safe spaces for adolescents to discuss SRHR issues and engaging adolescents in coming up with the solution.

**Conclusion:**

This study provides significant insight on the important roles that teachers CBHWs can play in addressing adolescents SRHR related problems. Overall, the study emphasizes the need to fully engage adolescents in addressing adolescents SRHR problems.

## Plain Language Summary

Comprehensive sexuality education programmes are often not implemented properly because facilitators are not adequately prepared, and the community usually resist such programs. Similarly, in Zambia, the teachers and CBWHs implementing sexual and reproductive health activities often felt uncomfortable discussing sensitive sexuality topics with adolescents. This study was conducted within a bigger research project exploring whether teachers and community-based health workers together can effectively deliver sexual and reproductive health information at school and community levels. Discussion on delivery ASRHR services were held with teachers and CBHWs to identify their roles, and challenges they experienced, and find solutions to problems. The interviews showed that the teachers and CBHWs provided sexual reproductive health and rights (SRHR) counselling to adolescents and parents. They were also involved in mobilising communities to attend SRHR meetings, and sensitise, and refer them to SRHR services. However, Teachers and CBHWs encountered several challenges. These include late reporting and hiding of sexual abuse cases, myths about contraceptives, and stigmatisation of girls with history of sexual abuse, and pregnancy. Thus, girls felt shy to participate in SRHR discussions due to customary norms and values regarding marriage. More community engagement opportunities are needed to break the barriers of communication, shift cultural norms to help enhance adolescent uptake of SRHR services in order prevent pregnancy and other related challenges.

## Introduction

Poor of access to sexual reproductive health and rights services (SRHR) continue to be one of the leading contributors to the burden of SRHR related problems such as sexual assault, rape, and early pregnancies among adolescents [1]. In sub–Saharan Africa, adolescent girls and young women remain as one of the sub-population most susceptible to unplanned pregnancies, early marriages, sexual violence, and HIV infections [2, 3]. For example, in Zambia adolescents and young people currently constitute more than half of the total population and yet, they also face many health and SRHR related problems which impact their lives and in the long run impacting on the overall health status of the country [4]. Although adolescents often are encouraged to delay sexual activity until marriage or until they are more mature, surveys show that 50% of Zambian adolescents have had sex before they reach their 18th birthday. This could be attributed to early marriages, traditional beliefs and values on sexuality [4]. Research indicates that myths around contraceptive coupled with existing barriers in obtaining contraceptives and lack of communication between partners may lead many adolescents to have unprotected sex than to use effective protection, putting them at risk of sexually transmitted infections and early pregnancy [2]. Thus, it is important that adolescents are properly informed about how they can protect themselves and make informed decisions on their sexuality. Teachers and CBHWs are essential in equipping adolescents about SRHR because they interact with in and out of school adolescents on a regular basis. Further, their involvement is important because they are also considered as role models who can help in addressing the sensitivity and taboos associated with SRHR.

## Response adolescent, sexual, reproductive, health and rights in Zambia

Preventing adolescent pregnancy and marriage is on the socio-economic and political agenda in Zambia. In the last decade, the government of the republic of Zambia has introduced several programs including integration of comprehensive sexuality education (CSE) in schools to improve SRHR information among school and non-school going adolescents and young adults. In 2014, the government of Zambia completed the development of a CSE framework, and officially rolled it out to all schools, targeting pupils aged 10–18 in grades 5–12 of schooling. Comprehensive Sexuality Education was integrated in carrier subjects such as science, social studies, and religious education and teachers are expected to deliver CSE as they teach the carrier subjects [5]. Zambia’s CSE curriculum covers six thematic areas: relationships; values, attitudes, and skills; culture, society, and human rights; human development; sexual behaviours, and ASRHR. The overall goal of CSE is to equip adolescent and young people with knowledge for them to make informed decisions on the sexuality, to enjoy better sexual and reproductive health and have better health outcomes overall [5].

Implementation of CSE in both schools and communities has not been without challenges. Firstly, engagement of parents and stakeholders has been limited, impacted by resistance, and normative discourses that un-married adolescents are too young to discuss sexual related issues [6, 7]. Adolescents especially girls in Zambia still face challenges early marriages, pregnancy, and school dropout. The Research Initiative to Support the Empowerment of Girls (RISE) intervention was implemented across 150 schools in Zambia from 2016 to 2020 to test interventions for reducing early marriages and school dropout.

The RISE trial was implemented in 12 selected districts of Southern and Central Provinces of Zambia. RISE was a cluster community randomized controlled trial implemented by the University of Zambia in collaboration with the University of Bergen and Chr. Michelsen Institute (Norway) [6, 8, 9]. One of the objectives of the RISE was to measure the effectiveness of a combined economic and community intervention on childbearing, early marriage and school dropout [10]. The RISE project had 3 interventions arms, but the focus of this study is the third arm which was commonly referred to as the community intervention arm. Details of the Community intervention arm of RISE project and assumptions related to how and under what circumstance the intervention could work (or not) are documented elsewhere [8]. The community intervention arm included: community and parents’ involvement in promoting supportive social norms around postponement of early marriage and early childbearing as well as promoting education and SRHRs for girls. The key actors in the community setting arm: teachers and CBHWs who conducted community meetings on sexual reproductive health and rights with parents and guardians in the communities [6, 10]. From 2016 to 2018, RISE youth club meetings were also held after regular school hours for girls and boys who were enrolled in grade 7 in 2016 and they were welcome to continue to participate in the youth club for the 2 years duration even if they dropped out of school. The aims of the youth club were to provide adolescents with knowledge about sexual and reproductive health, including modern contraceptives, clarify misconceptions and myths, change beliefs relating to usage of contraceptives; and to empower young people to make good decisions, improve their ability to communicate about ASRHR with partners, and their ability to negotiate about condoms and contraceptives. Similar topics were discussed during community meetings targeting parents of the RISE participants and interested community members [6, 11].

Several challenges and opportunities have been documented on implementing SRHR in youth clubs within the school settings [6, 12–14]. However, little evidence exists around experiences of teachers and CBHWs` promoting the prevention adolescents’ sexual reproductive health and rights problems in community settings. Therefore, this study sought to explore teachers and CBHWs’ experiences in addressing adolescents’ sexual reproductive health and rights problems in rural health **systems** within the context of the RISE project.

## Methodology

### Study design

This study employed a qualitative narrative inquiry study design. This design is suitable for this study in that it seeks to get in-depth understanding of the subject matter through documenting significant experiences of the people involved in implementing interventions [15]. In the context of this study, adoption of the narrative approach enabled the teachers and CBHWs to share detailed information about their day-to-day experiences about working with adolescents and young people in delivering SRHR services in rural health systems. Further, the narrative approach was a suitable design for this study because it helped reveal unique perspectives and deeper personal explanations or narrations of the experiences. Narrative inquiry also provides in-depth understanding on chosen moments of significance in this case the experiences of teachers and CBHWs as they addressed ASRHR problems in community meetings and youth clubs of the RISE project [13]. All methods were carried out in accordance with relevant guidelines and regulations for conducting qualitative research [16].

### Study population and participants

This study was conducted in purposely sampled schools in central province of Zambia, under the community component arm of the RISE project. Fifteen (15) schools in the community component arm of the RISE project across four districts of Central Province namely Kabwe, Chibombo, Chisamba and KapiriM-Mposhi districts were selected. At each of the schools, one teacher and one CBHW, in charge of organizing the RISE project activities described above were interviewed. Among the 15 selected schools, 10 had the lowest prevalence of SRHR challenges while five (5) has reported the highest prevalence of SRHR challenges such as adolescent marriages, pregnancies and school drop out after the RISE project was under implementation for at least 2 years.

### Data collection methods, and sampling strategy

Data collection took place between August and December 2018. Maximum-variation sampling criteria was used to include a mix of teachers and CBHWs from both semi urban and rural schools. Maximum variation helped to narrow down to the suitable group because it allowed for selection of specific participants in terms of location and type of problem handled and how frequently the school recorded any of the SRHR problems of interest. This brought the sample size of eligible participants to 22. Although attempts were made to contact all the 22 eligible participants for the interview, only 21 were available as one CBHW could not be reached. Therefore, a total of 21 interviews were conducted. Sixteen (16) out of the 21 participants were women (9 teachers and 7 CBHWs) and five were men (f4 teachers and 1 CBHW) as shown in Table 1 below. Further, 12 interviews were conducted face-to-face at the school premises, while the 9 were only available by phone. The interviews took approximately 40–60 min. The interviews were conducted using a mix of English, the official school language and Bemba, the local language in the communities where the study was conducted.

**Table 1:** Participants Information

District	Teacher	CBHWs	Male	Female	Number of Schools	Total
Chisamba	2	2	-	4	4	4
Chibombo	3	3	-	6	3	6
Kabwe	1	-	1	-	1	1
Kapiri-mposhi	3	2	2	3	3	5
Mkushi	4	1	3	2	4	5
Total	13	8	6	15	15	21

### Data management and analysis

The interviews were tape recorded and later transcribed verbatim by the first author (KC) and with close discussion with the second author (CM). To enhance the data quality, frequent discussion was held during data collection to discuss interview summaries with the all the authors. Thematic analysis was used to identify, analyse, and report pattern participants roles, challenges experienced, and solutions to deliver SRHR services. The first step in data analysis was reading and re-reading through the transcript to identify the initial codes. Preliminary codes were then discussed with all the other authors to come up with preliminary themes. Finally, after the robust discussion of the codes and re-reading of transcripts, the preliminary themes were consolidated into the final themes presented in this article. The coding process was carried out with the help of the qualitative software, NVivo version 12.

## Results

This study identified three themes: roles, challenges, and opportunities in relation to teachers and CBHWs experiences in promoting the prevention of ASRHR problems in rural health system in Zambia. The key findings are summarised in the Table 2 below.

**Table 2:** teachers and CBHWs roles, challenges, and opportunities in promoting the prevention of ASRHR problems in Zambia

*S/N*	*Major Themes*	*Codes*
***1***	The role of teachers and CBHWs in addressing sexual reproductive health problems	a. *Mobilizing and sensitizing the community for meetings* b. *Providing SRHR counseling services to both adolescents and guardians* c. Tactfully addressing misconceptions on SRHR services d. Brokering relationships between guardians and adolescent and the school e. Creating referral to other SRHR services if needed f. Follow-up of adolescents who do not report for school
***2***	Challenges experienced in the delivery of SRHR information and services	a. Stigmatization associated with difficult experiences such as sexual abuse and pregnancy b. Shyness among girls to participate when discussing SRHR in the presence of the boys c. Unreported and late reporting of sexual abuse cases d. Myths about contraception e. Customary norms and values regarding marriage affect SRHR information
***3***	Suggested strategies for addressing the challenges	a. Creating safe spaces for adolescents to discuss SRHR issues b. Engaging adolescents in coming up with the solution

### The role of teachers and CBHWs in addressing sexual reproductive health problems in rural health systems

#### Mobilizing and sensitizing the community during ASRHR meetings

The teachers and the community-based health workers (CBHWs) helped to mobilize the parents/guardians for the community meetings. Mobilization was done mostly by informing parents about the RISE related activities including inviting them to community meetings when parents came for the Parent Teacher Association (PTA) meetings in the schools. It was important parents/guardians to be invited to ASRHR meetings as they play a vital role in in influencing participation and uptake of reproductive health information and services among adolescents. As one participant said:

*“The CBHW at this school was always there to mobilize the parents for the community meetings, so that we can talk to them and address all concerns they may have****” (Teacher, Female****)*.

#### Providing SRHR counseling services to both adolescents and guardians

One other role that the teachers and CBHWs played was counselling adolescents and parents/guardians. In a situation where an adolescent got pregnant or tested HIV positive, the parents and the adolescent were for example taken through a process of counseling to prepare them on how to manage the new health condition and related challenges. According to some participants, providing counselling was important because most parents would react negatively to adolescents who found themselves pregnant or tested HIV positive, by scolding, shouting and chasing the girl from home. Counselling helped parents to manage the health challenge by equipping them with comprehensive knowledge of the reproductive health challenge and skills of managing it.

“*So, what the teacher and I do is to follow the girls up and see how they can be helped. When the teacher is busy, I go with the peer educator. When we get there, we start by counseling the guardians, who we come to learn somewhat contribute to the dropping out of school for these girls because they shout at the girls till they feel guilty and ashamed to go to school. We also talk to the girls on the importance of going back to school, to better her life and that off her child. So that is how I get involved in dealing with SRH problems. (CBHW, female).*

#### Tactfully addressing misconceptions on SRHR services

Participants also helped in addressing adolescents SRHR problems by clarifying misconceptions and skeptics that parents had regarding SRHR information and services. Clarification and information was done using manuals which were developed by the project that contained topics on how to address various ASRHR challenges. The main aim of address misconception was to promote positive values towards adolescent sexuality. Value clarification was done through community meetings and youth clubs. One of the issues that was clarified was about contraceptive use as most of the parents believed that the adolescents are too young to be using contraceptives and that use of such contraceptives would make the adolescents fail to have children in future. Teachers and CBHWs provided evidence-based information to address misconceptions, as one participants said:

*“…apart from dealing with the Adolescents and the SRHR related problems, we from time to time included the parents in the community meetings to help address the misconceptions and reservations that they had towards the project and the topics shared (Teacher, Female).*

#### Brokering relationships between guardians and adolescent and the school

The teachers and the CBHWs ensured a coordinated system between the school, homes, and clinics. Creating this relationship was important because barriers to addressing ASRHR problems such as early pregnancy and unsafe abortion were worsened by lack of proper coordination among the school, homes and clinics. This limited coordination hampered the proper flow of information on the benefits of SRHR information and services which resulted into limited support by parents and schools towards adolescents to access such information and services in the health facilities. Therefore, creating a good relationship between the parents and the adolescents and the school would create a supportive and conducive environment for adolescents to take up SRHR information and services in the health facilities and schools. Specifically, coordination between the school and the health facilities was enhanced by CBHWs who worked both at the school and the clinic. One of the participants described her coordination role with parents as follows:

*“Sometimes what used to work as solution would be to call parents through the head teacher’s office especially those who insisted on marrying off their children to help them understand if they know and think that their actions really benefit their children*…*sometimes even the boy responsible and his parents were invited”* (Teacher, female).

#### Creating referral to other SRHR services if needed

Respondents indicated that they worked closely with the clinics through the community-based health workers (who as mentioned above were also affiliated to the local clinic) to address the more sensitive problems like STIs and abortion by referring them to relevant institutions once identified. Most of the participants said that they referred problems such as sexual abuse to law enforcement agencies. Given that most learners prefer hiding these SRH problems due to their sensitivity, involvement of teachers and CBHWs facilitated referral processes because learners were free to open up to them when faced with SRHR challenges such as sexual abuse. One teacher described how she had stepped in to health the girls to access treatment for STIs as follows:

*“I discovered one girl who was said to have been sick for a long time, had an STI and her parents didn’t take her to the hospital until we stepped in to help the girl after follow ups and the girl was getting really sick with swollen genitals and a bad body odor, we quickly referred her to the clinic where she was attended to quickly….we actually heard that she had been taken to a witch doctor for healing but it didn’t work” (Teacher, Male).*

#### Follow-up of adolescents who miss school lessons

In cases were the teachers and community-based health workers noticed that some girls were absent from school they followed them up in the community to find out what was keeping them away from school. If absenteeism was due to a reproductive health challenge such as pregnancy, the teachers and community-based health workers encouraged and counseled the learners to report back to school, and further provided them information on how to manage or copy with the challenge both in the school and community.

*“We made sure to follow them up in the communities to really find out the reason why they stopped coming to school, because we realized trying to contact them by phone was not working and when they realize it is us trying to contact them, they would cut the line…so we resolved to following them up physically* (CBHW, male).

### Challenges experienced in the delivery of SRHR information and services

#### Stigmatization against girls with sexual abuse and pregnancy

Teachers and CBHWs reported that mocking of those who were pregnant or victims of sexual abuse by other adolescents made the affected adolescents to close and shun SRH information and services in the schools and health facilities. It was also reported that affected adolescents were often called names and laughed at by other students which made the adolescents to exclude themselves from school activities including SRHR youth clubs. Further, this stigma and discrimination affected the self-esteem, and school performance resulting into absenteeism and school dropping. Stigmatization also made it difficult for teachers and CBHWs to convince adolescents to resume school.

*“the challenge I faced with that girl was when she would come to school, her fellow pupils would laugh at her that used to make her abscond from school. So, to convince her and her mother to allow her to be coming to was really challenging for me” (Teacher, female).*

#### Shyness among girls to participate when discussing SRHR in the presence of the boys

At the beginning of the youth clubs in the schools, most respondents narrated that some adolescents especially girls did not participate fully in the youth club meetings in the company of boys. It was observed that during the sessions girls seemed to close up to the teachers and CBHWs, thus they did not get the required help to address their sexual reproductive health and rights related problems. One teacher narrated that in most cases adolescents were not opening easily especially those that experienced SRHR problems that are considered “socially sensitive” or embarrassing or shameful like having an STI or HIV.

*When we first introduced boys to youth clubs, I realized most girls would not participate fully in the discussions (Teacher, female).*

#### Unreported and late reporting of sexual abuse cases

Most respondents reported that some adolescents could not report cases of sexual abuse while others reported such cases but late. This posed as a challenge to the teachers because they were in most cases incapable of addressing the problem of sexual abuse in a timely manner.

*“looking at our community, in cases of rape and defilement, they usually do not cooperate with us from the school. Instead, they will try to cover up the truth, which poses as a challenge for us to help or play our role fully” (Teacher, Female).*

*“…No, we couldn’t help the girl because, after taking to the parents, they said we should not go to the police, because both parties refused to pursue the matter and destroyed the physical evidence, so we felt like our hands were tied.” (CBHW, male)*.

#### Myths about contraception’s

One of the most challenging components that the participants recorded was that most of the rural communities where this intervention was implemented have a lot of myths and misconceptions on contraceptives and contraceptive use. As already outlined, mixed opinions and concerns regarding girls’ accessing contraceptives, on the assumption that there are too young and exposing them to contraceptives at a young age might have severe reproductive health complications in future.

*after talking to the girls about contraceptives, one of the girls went and told their parent and that parent got very furious and said that it was not right for girls her age to be taught about contraceptives…After the meeting, the concerned parent asked me why I was talking to young girls about different types of contraceptives. A lot of parents were not for the idea, and for such I received a lot of criticism. (Teacher, female)*

#### Customary norms tensions against ASRHR education

Many girls continued to stop schooling because of early marriages. Customary norms such as the issue of parents marrying off their daughters early despite attending the community meetings worked against the teachings by teachers on the importance schooling. Despite all the lesson against child marriages discussed in the ASRHR community meetings, some parents still preferred to have their children stop school in preference for marriage. Further, more respondents also noted that these cultural practices that promoted early marriages increased risks of teenage pregnancies and STIs infection.

*“One parent said that, in my community those that refuse to marry off their girls are considered rebellious and are threatened to be chased form the village for going against the rules of the chiefdom and against cultural beliefs” (CBHW, Male).*

### Suggested strategies for addressing the challenges

#### Creating safe spaces for adolescents to discuss SRHR issues

Adolescent girls who experienced child abuse or rape felt embarrassment, and shame SRHR education including counselling. It is for this reason the youth club meetings created an environment where SRHR learningcould take place without being judgments. The adolescents were free to ask questions and share experiences without fear of being judged or criticized. This helped them develop trust and strong bonds such that the girls were encouraged to approach the teacher or the CBHW regarding their experiences in relation to sexual abuse.

*“I created really good relationships with some of these girls and they were like my sisters and they could come to me for anything and most of them even sneaked to the clinic whenever they could for any SRHR problem or clarification and question they may face” (CBHW, Female).*

*“Even after we had helped her seek medical help for the STI she had concealed for a longtime but I realized the girl still needed my counsel and help in many ways, so I tried by all means to stay in touch with her, I kept touch in with her to really help her even in my personal time because I realized how important this was for her (Teacher, female).*

#### Engaging fellow adolescents in delivery SRHR services

overcoming hostile environment in discussing sensitive SRHR services require peer educators and other stakeholders’ involvement. Most participants indicated that recruiting and working with their fellow adolescents to provision enhanced counselling. Peer educators through counselling helped some girls experiencing SRHR challenges to find solutions to the problems they encountered. The peer promoters also worked with the teachers and CBHWs to make follow ups on fellow adolescents affected by SRH challenges from their homes. In addition, the peer educator who had performed well academically also acted as role models for school-going adolescents. This motivated and created a desire in the adolescents to continue with school despite falling pregnant or being married early.

*“There was an instant where we asked a few girls to help us talk (counsel) to their friends, and encourage them to continue coming to school even if they may be pregnant and it seemed to have worked because the girls came back to school and even managed to write the exam (CBHW*, female).

## Discussion

This study explored experiences of teachers and community-based health workers ‘in relation their roles, challenges, and opportunities in addressing adolescent sexual, reproductive, health and rights problems in rural health systems in Zambia. Teachers and CBHWs assumed including mobilization of the community actors including parents to promote the delivery of SRHR services to adolescent is essential to ensure acceptability of the intervention. This study found that teachers, CBHWs and parents often supported each other in delivery of SRHR information. Studies conducted in Zambia and Ethiopia found that some parents may resist and oppose school-based actors to discuss issues on SRHR such as contraceptives and condoms to their children [12, 17]. Moreover, discussion on sexuality is incompatible to religious and cultural practices [18].

The study also found teachers and CBHWs were *providing SRHR* counselling services to both adolescents and guardians. This also contributed to strengthening referral in delivery SRHR services. Other studies in Africa have shown that teachers and CBHWs play a critical role in teaching pupils on issues of SRHR [19]. They act as bridge between parents and children to facilitate topics which parents are uncomfortable to talk about. However, some teacher also find it challenge to discuss sensitive topics to young people. Some teachers have not received any training on SRHR [20, 21]. Hence involvement of CBHWs is key to ensure that they comprehensively deliver these services to young people [6]. Adolescent and young people may also not be comfortable and freely discuss SRHR with any of these facilitators due to the social scripts and discourses sounding adolescent sexuality in Zambian [7]. Adolescent with a history of child pregnancy, marriages and abuse may feel stigmatized to social values [22]. Therefore, better strategies and interventions are needed to break the cultural and religious barriers affecting adolescents and young people for them to optimally access SRHR services.

The study also identified strategies that can enhance community actors’ responsiveness to adolescents SRHR needs. Participants reported for creating safe spaces for adolescents to discuss SRHR issues including sensitives topics. This could work for discussion general SRHR issues and if tailored could be use to reach adolescents who have been sexually abused. The non-judgmental environment is essential for young people to access information and services they need. Study conducted in Pakistan found that creating adolescent and young people safe SRHR services contributed to optimal access and utilization of SRHR services [17, 23]. The lack of such spaces has been found to a barrier contributing to many adolescents fail to access SRHR services. Therefore, adolescent friendly approaches must be used to engage adolescents to determine and identify not only the best approaches but also appropriate spaces for them to access SRHR service. This study found that the use of peer educators was acceptable and worked well to reach adolescents. Similarly, studies conducted in Uganda, Ethiopia and Ghana also confirmed that adolescent involvement in delivery SRHR is key in improving access and utilization of services [24–26]. This promotes collective action and ownership of the problem and solutions in addressing SRHR challenges including teenage pregnancies and marriages [26].

Implementation of SRHR interventions, therefore, requires collective action and collaboration of key community health systems actors including CBHWs, teachers, community leaders to ensure they integrate the training and SRHR service delivery in different community settings [6, 27–31]. This is also critical in ensuring that young people can easily access SRH services of their choice within the community context from stakeholders they trust [32]. However, very few countries ensure that relevant stakeholders are involved in the implementation SRHR intervention including CSE [21]. In this study, the collaboration between the teachers, parents and school seem to encourage adolescents to participate in youth clubs and access SRHR services when and if needed. Collaboration of the community actors beyond teachers and CBHWs to include traditional, and religious leaders, peer educators, and key stakeholders is critical to shape the prevention of the problem. The collaboration can also enhance strengthen referral mechanism of adolescent SRHR service providers. However, this can only work if: the provider for SRHR services is adolescents friendly; counselling is done through one-on-one sessions; group talks and presentations, and distribution of adolescents targeted print materials is done. In addition, peer involvement in the delivery of SRHR services remain an important strategy for youth participation in SRHR [22].

## Conclusion

This study provides significant insight on the important roles that teachers CBHWs can play in addressing adolescents SRHR related problems. The teachers and CBHWs played a key role roles in delivery of SRHR services through community mobilization, sensitization, counselling and referring adolescents and young people health facility to access services. Overall, the study emphasizes the need to fully engage adolescents in addressing adolescents SRHR problems. However, cultural norms towards access SRHR services made adolescents with pregnancy and those infected STDs to feel judged and shun from school and health services. Consequently, affected them from partcipating actively in SRHR services programmes. Collaboration with peer educators in delivery of SRHR education and counselling in local health systems either at school, homes and health facilities was found to be useful to increase the use of services among adolescent with SRHR challenges. Teachers, CBHWs and Peer educators were more likely to be trusted and thus made their adolescents comfortable and discuss freely issues that affected. Future studies could be conducted to further explore the role peer educator in shaping adolescents’ access to SRHR in rural health systems.

## Data Availability

The audio recordings and transcripts used and analyzed during this study are available from the corresponding author on reasonable request.

## Abbreviations

CBHS: Community Based Health Systems
CBHWs: Community Based Health Workers
FP: Family Planning
GBV: Gender Based Violence
HIV: Human Immune Virus
IDIs: In-depth Interviews
LMIC: Low and Middle Income Counties
RISE: Research Initiative to Support Empowerment of Girls
STI: Sexually Transmitted Infections
SRHR: Sexual Reproductive Health and Rights
UNESCO: United Nations Education Scientific and Cultural Organization
UNFPA: United Nations Population Fund
WHO: World Health Organization
